# HIV-1 Tat and Viral Latency: What We Can Learn from Naturally Occurring Sequence Variations

**DOI:** 10.3389/fmicb.2017.00080

**Published:** 2017-01-30

**Authors:** Doreen Kamori, Takamasa Ueno

**Affiliations:** ^1^Center for AIDS Research, Kumamoto UniversityKumamoto, Japan; ^2^International Research Center for Medical Sciences, Kumamoto UniversityKumamoto, Japan

**Keywords:** HIV-1, Tat, latency, transactivation, variability, reactivation

## Abstract

Despite the effective use of antiretroviral therapy, the remainder of a latently HIV-1-infected reservoir mainly in the resting memory CD4^+^ T lymphocyte subset has provided a great setback toward viral eradication. While host transcriptional silencing machinery is thought to play a dominant role in HIV-1 latency, HIV-1 protein such as Tat, may affect both the establishment and the reversal of latency. Indeed, mutational studies have demonstrated that insufficient Tat transactivation activity can result in impaired transcription of viral genes and the establishment of latency in cell culture experiments. Because Tat protein is one of highly variable proteins within HIV-1 proteome, it is conceivable that naturally occurring Tat mutations may differentially modulate Tat functions, thereby influencing the establishment and/or the reversal of viral latency *in vivo.* In this mini review, we summarize the recent findings of Tat naturally occurring polymorphisms associating with host immune responses and we highlight the implication of Tat sequence variations in relation to HIV latency.

## Introduction

Viral latency is a reversible state whereby a pathogenic virus becomes dormant (latent) during the viral life cycle in individual cells. HIV-1 may either actively replicate to rapidly produce progeny virions or can enter a long-lived quiescent state (viral latency), from which it may later be subsequently reactivated. The mechanisms for establishment and maintenance of HIV-1 latency mainly operate at the transcriptional level by both viral (Yukl et al., 2009; Donahue et al., 2012; Donahue and Wainberg, 2013; Ranasinghe et al., 2013) and host (Coiras et al., 2009, 2010; Donahue and Wainberg, 2013) machineries and occur at the levels of transcription, chromatin modification, and epigenetic regulations (Coiras et al., 2009; Donahue and Wainberg, 2013; Archin et al., 2014; Cary et al., 2016).

HIV-1 latency is primarily found within resting memory CD4^+^ T cells (Chun et al., 1995, 1997; Dahabieh et al., 2015), microglia cells (Chakrabarti et al., 1991; Davis et al., 1992), monocytes/macrophages (Battistini and Sgarbanti, 2014; Kumar et al., 2014; Abbas et al., 2015), and others (Canki et al., 2001; MacDougall et al., 2002; Valentin et al., 2002) which intrinsically have a long half-life *in vivo*. Because the expression level of the viral proteins is absent or poorly expressed and also the existence of immune escape mutations (Deng et al., 2015), the latently infected cells are much less susceptible to be recognized and cleared by the host immune system, viral cytopathic effects or currently available antiretroviral drugs. Thus to date, latently infected viral reservoir is one of the fundamental limitations toward HIV cure (Marsden and Zack, 2015).

Among the viral proteins, HIV-1 Tat has attracted more attention in viral latency because it potently plays a role in viral transcription regulation. Structurally, Tat is a small nuclear protein with amino acid length ranging from 86 to 101 and the molecular weight ranging from 14 to 16 kDa (Ruben et al., 1989). Functionally, Tat is divided by six domains and plays a role in nuclear translocation (Efthymiadis et al., 1998; Rana and Jeang, 1999), binding for viral RNA (Roy et al., 1990), several host factors and co-factors (Jeang et al., 1993; Garber et al., 1998; Marzio et al., 1998), and the transactivation of 5′ long terminal repeat (LTR) (Ruben et al., 1989; Roy et al., 1990; Jeang et al., 1993; Tong-Starksen et al., 1993; Neuveut and Jeang, 1996). Despite such fundamental functions in the virus life cycle, Tat is a highly polymorphic protein comparable to other HIV-1 polymorphic proteins such as Env, Vpu, and Nef (Yusim et al., 2002; Rossenkhan et al., 2012). Recent studies indicate that a substantial part of viral polymorphisms including in Tat is caused by viral mutational escape from cellular immune responses (Allen et al., 2000; Mason et al., 2009; John et al., 2010; Carlson et al., 2012). It is conceivable that naturally occurring mutations in Tat may modulate transactivation or other Tat functions, and that consequently affect the establishment and reversal of HIV-1 latency *in vivo*. In this mini review, we will describe the role of HIV-1 Tat toward HIV-1 latency establishment and reactivation, and discuss the possibility that naturally occurring Tat mutations may influence viral latency. The details of host machinery in relation to HIV-1 latency have been well described in recent reviews (Ruelas and Greene, 2013; Dahabieh et al., 2015; Cary et al., 2016) and are not discussed here.

### The Role of HIV-1 Tat in Establishment of Viral Latency

**FIGURE 1:**
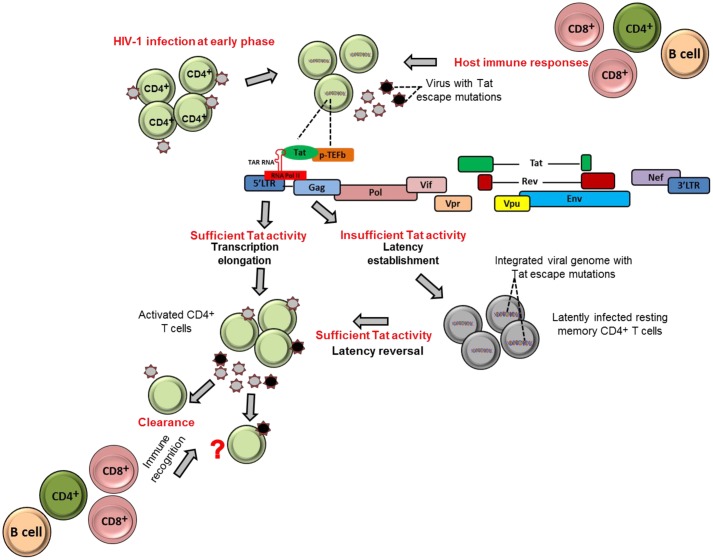
**Tat role in establishment and reversion of viral latency.** The schematic figures illustrate how HIV-1 Tat may contribute to establishment of viral latency and latency reversal in resting memory CD4^+^ T cells in HIV-1 infection.

Tat ensures high levels of viral transcription during the virus life cycle (Das et al., 2011). The protein stimulates transcription from the viral 5′ LTR promoter and controls RNA polymerase II (RNAP II) elongation. This is achieved by Tat binding to the TAR hairpin in the nascent RNA transcript and the complex of positive transcription elongation factor b (P-TEFb) composed of Cyclin T1 (CycT1) and cyclin-dependent kinase 9 (CDK9) which phosphorylates the C-terminal domain of the RNAP II that consequently promote transcriptional elongation from the viral promoter (**Figure 1**) (Dahmus, 1996; Parada and Roeder, 1996; Das et al., 2011; Peterlin et al., 2012). Importantly, the absence or inactivation of Tat in HIV-1 infection has been observed to predominantly generate short non-polyadenylated transcripts of less than 100 nucleotides in length that forms the TAR stem-loop structure, and resulted in reduction of viral transcription and replication (Feng and Holland, 1988; Roy et al., 1990; Yedavalli et al., 2003; Pagans et al., 2005; Das et al., 2011) (**Figure 1**).

It could be therapeutically beneficial if we could prevent or at least reduce to a large extent the size of the established latent reservoir. Evidence indicates that Tat, when present in sufficient quantities, may counteract the establishment of HIV-1 latency by promoting transcriptional initiation or elongation (Pearson et al., 2008; Donahue et al., 2012). One study demonstrated that fewer latently infected cells were established in Jurkat cells that stably express Tat compared to cells that did not express Tat (Donahue et al., 2012). These findings highlight the contribution of Tat and its abundance on prevention of establishment of viral latency. In contrast, a complete block of Tat activity may induce permanent latency as observed with use the of Tat dependent transcription inhibitors such as didehydro-cortistatin A (dCA). The agent has been shown to induce permanently the inactivation of the viral transcription in primary latently infected CD4^+^ T cells isolated from aviremic ART-treated subjects; and also when tested in several cell line models of latency (HeLa-CD4, promyelocytic OM-10.1 and J-Lat T-lymphocytic cell lines) (Mousseau et al., 2015). In addition, in the same study both in primary cells and latently infected cell line models, the dCA established a state of latency with an extremely impaired ability to reactivate even in the presence of conventional latency-reversing agents (such as TNF-α and prostratin). Therefore, the concomitant treatment of dCA and antiretroviral drugs may reduce the size of reactivation of latently infected cells *in vivo* and eventually attain a functional HIV cure. However, to date, most experiments done for dCA are limited to *in vitro* models of latently infected cell lines and primary CD4^+^ T cells. Therefore, further studies are needed to test the efficacy and safety of dCA as a viral transcription inhibitor agent in advanced experimental systems such as using humanized mice and non-human primates.

### Role of Tat Protein on Reversion of Viral Latency

Tat can also contribute to reactivation of latently infected cells. For example, previous studies demonstrated that Tat is responsible for directly activating viral transcription in the patient-derived latently infected resting memory CD4^+^ T cells without requiring cellular activation (Lin et al., 2003; Lassen et al., 2006). This is also supported by the Jurkat model of latency showing that the introduction of exogenous Tat was sufficient to reactivate most of the latently infected population (Donahue et al., 2012). Similarly, HIV-1 latently infected cells, at least in Jurkat cells, can be reactivated by cellular superinfection in a Tat-dependent manner (Donahue et al., 2013). Moreover, both experimental and computational methods have revealed that Tat is more effective than cellular activation approaches in reactivation of full-length transcription of latent HIV. In a recent study, Razooky et al. (2015) showed that removal of cell activation stimuli in HIV-infected primary CD4^+^ T cells resulted in a drastic decline in cellular activation, but viral transcription activity as measured by GFP expression of productively infected cells remained relatively unchanged. Furthermore, the same study revealed by a computational method of HIV transcriptional modulation that Tat in abundance alone is sufficient for reactivation of the latently infected cells (Razooky et al., 2015). In addition, the depletion of some host factors or molecules that inhibit Tat transactivation activities, such as the long non-coding RNAs (NRON) that degrades Tat protein, in combination with a histone deacetylase (HDAC) inhibitor, has also been shown to significantly reactivate HIV-1 latency in CD4^+^ T lymphocytes (Li et al., 2016). Furthermore, in a recent mutational study, a Tat mutant, Tat-R5M4 that comprises of V36A, Q66A, V67A, S66A, and S77A mutations, exhibited a potent ability to reactivate latently infected CD4^+^ T lymphocytes (Geng et al., 2016). Taken together, these findings provide a potential alternative approach toward reactivation of the latently infected cells with Tat protein.

### Effects of Tat Variability on Latency

**FIGURE 2:**
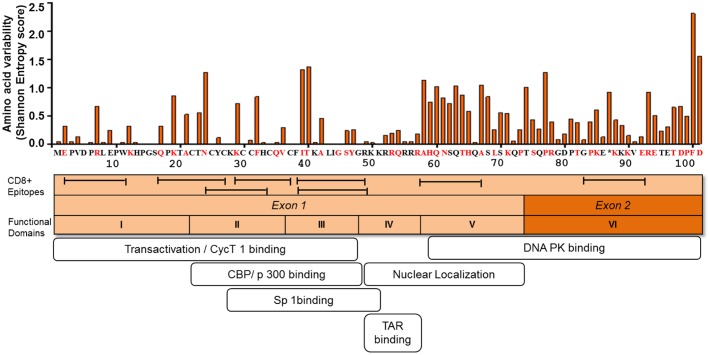
**Amino acid variability, immunogenic sites for CTL, and functionally important sites for transactivation activity of HIV-1 Tat.** The figure depicts the amino acid variability of Tat as measured by the Shannon entropy score for subtype B Tat sequences (*n* = 378) obtained from a public database (Los Alamos Sequence Database). The immune reactive sites are shown with location of CTL epitopes and the amino acid residues that are associated with CTL escape as determined by statistical association with host HLA class I allele (red). Tat_HXB2_ is used as reference. The domains associated with the transactivation activity are also indicated.

Sequence analysis of plasma viral RNA isolated from cross-sectional and longitudinal collection of HIV-infected individuals exhibited that HIV-1 Tat is a highly variable protein even among the rapidly mutating HIV-1 proteins such as Env, Vpu, and Nef (Yusim et al., 2002; Li et al., 2015). The high genetic variability of HIV-1 Tat is observed across the subtypes, such as subtypes B and C, in the major HIV-1 group M, and also across HIV-1 groups O and N as well as HIV-2 (Yusim et al., 2002; Rossenkhan et al., 2012; Li et al., 2015; Roy et al., 2015b). Interestingly, Bayesian evolutionary analysis model demonstrated that subtype B Tat has evolved relatively faster than other subtypes (Roy et al., 2015a). The extent of amino acid variability in Tat as estimated by the Shannon entropy score in subtype B sequences published in Los Alamos sequence database is illustrated in **Figure 2**.

Mutational studies of HIV-1 Tat revealed that Tat is divided into six functional domains (Kuppuswamy et al., 1989) (**Figure 2**). The first three domains are responsible for Tat transactivation activity and binding with the transcription cofactors (Feng and Holland, 1988; Feinberg et al., 1991; Garber et al., 1998; Wei et al., 1998; Rusnati et al., 1999); while the fourth domain is a TAR binding domain (Dingwall et al., 1989; Roy et al., 1990; Weeks and Crothers, 1991). The fourth and fifth domains are important for Tat nuclear localization (Ruben et al., 1989), the sixth domain binds to DNA PK and also contribute to viral infectivity (Smith et al., 2003). Importantly in regard to viral latency the functional domains II and III, spanning amino acid positions 22 to 48, are shown to be responsible for transactivation activity (**Figure 2**). The several mutations at positions 22 to 40 amino acid residues (including highly conserved cysteine residues) have been shown to be deleterious with respect to Tat transactivation activity; whereas those at positions 1 to 21 amino acid residues are relatively functionally tolerated (Kuppuswamy et al., 1989; Ruben et al., 1989). Tat plays active role in productive viral replication mainly through enhancement of transcription at viral LTR promoter. Mutational studies have shown there is a strong correlation between Tat transactivation activity and viral replication capacity, whereby the functionally defective Tat has ability to severely inhibit viral replication *in vitro* (Verhoef et al., 1997; Das et al., 2011). This suggests that provirus with functionally defective Tat influences the viral replication and size of the latent reservoir *in vivo*. In respect to the naturally occurring mutations from HIV-1-infected individuals, the Cys-22 to Ser mutation (C22S) in HIV-1 Oyi strain resulted in loss of transactivation activity and was enriched in long-term non-progressive patients (Huet et al., 1989; Peloponese et al., 1999; Watkins et al., 2006). Moreover, several naturally occurring polymorphisms, including P10S, W11R, K19R, A42V, and Y47H, that were observed in 5 HIV-infected subjects at acute or early infection stage, demonstrated impaired transactivation activity and were statistically significantly enriched in the latently infected CD4^+^ T cells (Yukl et al., 2009). These findings suggest that certain naturally occurring mutations can influence Tat transactivation activity and the establishment of viral latency or reactivation of latent reservoirs during the course of HIV-1 infection *in vivo*. Therefore, this issue warrants for more comprehensive study using a large number of HIV-infected subjects.

### Genetic Variability of Tat Driven by Immune-Mediated Selection Forces

It is becoming evident that mutational escape from CD8^+^ cytotoxic T lymphocyte (CTL) responses represents a potent ongoing driver of global HIV-1 diversification (Price et al., 1997; Goulder et al., 2001; Brumme et al., 2009; Carlson et al., 2012). Tat has also been shown to be frequently targeted by the host HLA-restricted CTL responses (Addo et al., 2001, 2002; Westrop et al., 2009). A number of CTL epitopes have been identified, including PW9 (^3^PVDPRLEPW^11^) and EW10 (^2^EPVDPNLEPW^11^) restricted by the protective HLA-I alleles, HLA-B^∗^57 and HLA-B^∗^5801, respectively (Schellens et al., 2008; Zhai et al., 2008; Chopera et al., 2011). Additional epitopes are well summarized at the web site, http://www.hiv.lanl.gov/content/immunology/maps/ctl/Tat.html. CTL epitopes are distributed in both highly conserved and polymorphic regions in Tat; however, more number of CTL epitopes are reported at the relatively conserved regions to date (**Figure 2**). A number of Tat mutations in both conserved and variable regions have been reported to be associated with host cellular immune responses in various viral subtypes and host populations (**Figure 2**) (Allen et al., 2000; Guillon et al., 2006; Mason et al., 2009; John et al., 2010; Carlson et al., 2012). Importantly, some of the CTL escape mutations in Tat such as F32L and V36S observed in a frequently recognized (or immunodominant) Tat epitope, CC8 (^30^CCFHCQVC^37^) restricted by HLA-C^∗^12:03 (Cao et al., 2003; Liu et al., 2007, 2011), are located at sites that are important for transactivation and co-factor binding (**Figure 2**). Some other CTL escape mutations are located at functionally important regions; N24K, N24T, K29R, and K29S in NF9 (^24^NCYCKRCCF^32^) epitope restricted by HLA-A^∗^29:02 (Jones et al., 2004), K40T in FY10 (^38^FQKKGLGISY^47^) restricted by HLA-B^∗^15:03 (Liu et al., 2013), and R7S, R7K, and E9D in PW9 (^3^PVDPRLEPW^11^) restricted by HLA-A^∗^25:01 (Liu et al., 2007). These data suggest that CTL escape mutations in Tat, especially those located at functionally important conserved regions, have a potential to differentially influence Tat activity. However, it remains elusive as to what extent CTL responses to Tat or CTL escape mutations in Tat may influence viral latency kinetics both at establishment and reversal stages. Also, it is intriguing to ask whether Tat mutations may influence immune recognition of latently infected cells after reactivation. It is also worth to mention that despite the predominant effect of CTL selection pressure on Tat sequence polymorphism, other host immune responses such as those mediated by CD4^+^ T cells (Lichterfeld et al., 2012; Ranasinghe et al., 2013) and B cells (Goldstein et al., 2001; Moreau et al., 2004) also target Tat; and may therefore potentially impose selection pressure leading to escape mutations which may differentially affect Tat activity.

## Conclusion and Future Perspectives

To date, the highly genetic viral variability and the existence of latently infected resting CD4^+^ T lymphocytes and other cells *in vivo* are among the setbacks toward achievement of complete HIV control and eradication. It is generally thought that virus can acquire mutations and evade host immune responses while maintain their fitness effects as minimal as possible. However, similar to the cases in the other HIV-1 proteins such as Gag (Goulder et al., 2001; Troyer et al., 2009) and Nef (Mwimanzi et al., 2013; Kuang et al., 2014), certain naturally occurring immune-associated mutations in Tat may impose fitness cost to the virus. However, it remains poorly described how immune-mediated Tat polymorphisms affect either establishment of viral latency or reactivation of the latently infected cells and also the consequence of such viral polymorphisms on immune recognition. These points could open a new venue to modulate HIV latency and reversal of latency *in vivo* for future therapeutic application toward cure.

## Author Contributions

DK and TU conceived, designed, compiled the data, and wrote the manuscript.

## Conflict of Interest Statement

The authors declare that the research was conducted in the absence of any commercial or financial relationships that could be construed as a potential conflict of interest.

## References

[B1] Abbas W. Tariq M. Iqbal M. Kumar A. Herbein G. (2015). Eradication of HIV-1 from the macrophage reservoir: an uncertain goal? *Viruses* 7 1578–1598. 10.3390/v7041578 25835530 PMC4411666

[B2] Addo M. M. Altfeld M. Rosenberg E. S. Eldridge R. L. Philips M. N. Habeeb K. (2001). The HIV-1 regulatory proteins Tat and Rev are frequently targeted by cytotoxic T lymphocytes derived from HIV-1-infected individuals. *Proc. Natl. Acad. Sci. U.S.A.* 98 1781–1786. 10.1073/pnas.98.4.1781 11172028 PMC29334

[B3] Addo M. M. Yu X. G. Rosenberg E. S. Walker B. D. Altfeld M. (2002). Cytotoxic T-lymphocyte (CTL) responses directed against regulatory and accessory proteins in HIV-1 infection. *DNA Cell Biol.* 21 671–678. 10.1089/104454902760330219 12396610

[B4] Allen T. M. O’Connor D. H. Jing P. Dzuris J. L. Mothe B. R. Vogel T. U. (2000). Tat-specific cytotoxic T lymphocytes select for SIV escape variants during resolution of primary viraemia. *Nature* 407 386–390. 10.1038/35030124 11014195

[B5] Archin N. M. Sung J. M. Garrido C. Soriano-Sarabia N. Margolis D. M. (2014). Eradicating HIV-1 infection: seeking to clear a persistent pathogen. *Nat. Rev. Microbiol.* 12 750–764. 10.1038/nrmicro3352 25402363 PMC4383747

[B6] Battistini A. Sgarbanti M. (2014). HIV-1 latency: an update of molecular mechanisms and therapeutic strategies. *Viruses* 6 1715–1758. 10.3390/v6041715 24736215 PMC4014718

[B7] Brumme Z. L. John M. Carlson J. M. Brumme C. J. Chan D. Brockman M. A. (2009). HLA-associated immune escape pathways in HIV-1 subtype B Gag, Pol and Nef proteins. *PLoS ONE* 4:e6687 10.1371/journal.pone.0006687 PMC2723923 19690614

[B8] Canki M. Thai J. N. Chao W. Ghorpade A. Potash M. J. Volsky D. J. (2001). Highly productive infection with pseudotyped human immunodeficiency virus type 1 (HIV-1) indicates no intracellular restrictions to HIV-1 replication in primary human astrocytes. *J. Virol.* 75 7925–7933. 10.1128/JVI.75.17.7925-7933.2001 11483737 PMC115036

[B9] Cao J. McNevin J. Malhotra U. McElrath M. J. (2003). Evolution of CD8^+^ T cell immunity and viral escape following acute HIV-1 infection. *J. Immunol.* 171 3837–3846. 10.4049/jimmunol.171.7.3837 14500685

[B10] Carlson J. M. Brumme C. J. Martin E. Listgarten J. Brockman M. A. Le A. Q. (2012). Correlates of protective cellular immunity revealed by analysis of population-level immune escape pathways in HIV-1. *J. Virol.* 86 13202–13216. 10.1128/JVI.01998-12 23055555 PMC3503140

[B11] Cary D. C. Fujinaga K. Peterlin B. M. (2016). Molecular mechanisms of HIV latency. *J. Clin. Invest.* 126 448–454. 10.1172/JCI80565 26731470 PMC4731164

[B12] Chakrabarti L. Hurtrel M. Maire M. A. Vazeux R. Dormont D. Montagnier L. (1991). Early viral replication in the brain of SIV-infected rhesus monkeys. *Am. J. Pathol.* 139 1273–1280. 1750503 PMC1886455

[B13] Chopera D. R. Mlotshwa M. Woodman Z. Mlisana K. de Assis Rosa D. Martin D. P. (2011). Virological and immunological factors associated with HIV-1 differential disease progression in HLA-B 58:01-positive individuals. *J. Virol.* 85 7070–7080. 10.1128/JVI.02543-10 21613398 PMC3126593

[B14] Chun T. W. Carruth L. Finzi D. Shen X. DiGiuseppe J. A. Taylor H. (1997). Quantification of latent tissue reservoirs and total body viral load in HIV-1 infection. *Nature* 387 183–188. 10.1038/387183a0 9144289

[B15] Chun T. W. Finzi D. Margolick J. Chadwick K. Schwartz D. Siliciano R. F. (1995). In vivo fate of HIV-1-infected T cells: quantitative analysis of the transition to stable latency. *Nat. Med.* 1 1284–1290. 10.1038/nm1295-1284 7489410

[B16] Coiras M. Lopez-Huertas M. R. Perez-Olmeda M. Alcami J. (2009). Understanding HIV-1 latency provides clues for the eradication of long-term reservoirs. *Nat. Rev. Microbiol.* 7 798–812. 10.1038/nrmicro2223 19834480

[B17] Coiras M. Lopez-Huertas M. R. Sanchez del Cojo M. Mateos E. Alcami J. (2010). Dual role of host cell factors in HIV-1 replication: restriction and enhancement of the viral cycle. *AIDS Rev.* 12 103–112. 20571604

[B18] Dahabieh M. S. Battivelli E. Verdin E. (2015). Understanding HIV latency: the road to an HIV cure. *Annu. Rev. Med.* 66 407–421. 10.1146/annurev-med-092112-152941 25587657 PMC4381961

[B19] Dahmus M. E. (1996). Phosphorylation of mammalian RNA polymerase II. *Methods Enzymol.* 273 185–193. 10.1016/S0076-6879(96)73019-7 8791612

[B20] Das A. T. Harwig A. Berkhout B. (2011). The HIV-1 Tat protein has a versatile role in activating viral transcription. *J. Virol.* 85 9506–9516. 10.1128/JVI.00650-11 21752913 PMC3165771

[B21] Davis L. E. Hjelle B. L. Miller V. E. Palmer D. L. Llewellyn A. L. Merlin T. L. (1992). Early viral brain invasion in iatrogenic human immunodeficiency virus infection. *Neurology* 42 1736–1739. 10.1212/WNL.42.9.1736 1513462

[B22] Deng K. Pertea M. Rongvaux A. Wang L. Durand C. M. Ghiaur G. (2015). Broad CTL response is required to clear latent HIV-1 due to dominance of escape mutations. *Nature* 517 381–385. 10.1038/nature14053 25561180 PMC4406054

[B23] Dingwall C. Ernberg I. Gait M. J. Green S. M. Heaphy S. Karn J. (1989). Human immunodeficiency virus 1 tat protein binds trans-activation-responsive region (TAR) RNA in vitro. *Proc. Natl. Acad. Sci. U.S.A.* 86 6925–6929. 10.1073/pnas.86.18.6925 2476805 PMC297963

[B24] Donahue D. A. Bastarache S. M. Sloan R. D. Wainberg M. A. (2013). Latent HIV-1 can be reactivated by cellular superinfection in a Tat-dependent manner, which can lead to the emergence of multidrug-resistant recombinant viruses. *J. Virol.* 87 9620–9632. 10.1128/JVI.01165-13 23804632 PMC3754111

[B25] Donahue D. A. Kuhl B. D. Sloan R. D. Wainberg M. A. (2012). The viral protein Tat can inhibit the establishment of HIV-1 latency. *J. Virol.* 86 3253–3263. 10.1128/JVI.06648-11 22238306 PMC3302319

[B26] Donahue D. A. Wainberg M. A. (2013). Cellular and molecular mechanisms involved in the establishment of HIV-1 latency. *Retrovirology* 10:11 10.1186/1742-4690-10-11 PMC3571915 23375003

[B27] Efthymiadis A. Briggs L. J. Jans D. A. (1998). The HIV-1 Tat nuclear localization sequence confers novel nuclear import properties. *J. Biol. Chem.* 273 1623–1628. 10.1074/jbc.273.3.1623 9430704

[B28] Feinberg M. B. Baltimore D. Frankel A. D. (1991). The role of Tat in the human immunodeficiency virus life cycle indicates a primary effect on transcriptional elongation. *Proc. Natl. Acad. Sci. U.S.A.* 88 4045–4049. 10.1073/pnas.88.9.4045 2023953 PMC51590

[B29] Feng S. Holland E. C. (1988). HIV-1 tat trans-activation requires the loop sequence within tar. *Nature* 334 165–167. 10.1038/334165a0 3386755

[B30] Garber M. E. Wei P. KewalRamani V. N. Mayall T. P. Herrmann C. H. Rice A. P. (1998). The interaction between HIV-1 Tat and human cyclin T1 requires zinc and a critical cysteine residue that is not conserved in the murine CycT1 protein. *Genes Dev.* 12 3512–3527. 10.1101/gad.12.22.3512 9832504 PMC317238

[B31] Geng G. Liu B. Chen C. Wu K. Liu J. Zhang Y. (2016). Development of an attenuated tat protein as a highly-effective agent to specifically activate HIV-1 latency. *Mol. Ther.* 24 1528–1537. 10.1038/mt.2016.117 27434587 PMC5113098

[B32] Goldstein G. Tribbick G. Manson K. (2001). Two B cell epitopes of HIV-1 Tat protein have limited antigenic polymorphism in geographically diverse HIV-1 strains. *Vaccine* 19 1738–1746. 10.1016/S0264-410X(00)00393-5 11166899

[B33] Goulder P. J. Brander C. Tang Y. Tremblay C. Colbert R. A. Addo M. M. (2001). Evolution and transmission of stable CTL escape mutations in HIV infection. *Nature* 412 334–338. 10.1038/35085576 11460164

[B34] Guillon C. Stankovic K. Ataman-Onal Y. Biron F. Verrier B. (2006). Evidence for CTL-mediated selection of Tat and Rev mutants after the onset of the asymptomatic period during HIV type 1 infection. *AIDS Res. Hum. Retroviruses* 22 1283–1292. 10.1089/aid.2006.22.1283 17209772

[B35] Huet T. Dazza M. C. Brun-Vezinet F. Roelants G. E. Wain-Hobson S. (1989). A highly defective HIV-1 strain isolated from a healthy Gabonese individual presenting an atypical western blot. *AIDS* 3 707–715. 10.1097/00002030-198911000-00004 2559749

[B36] Jeang K. T. Chun R. Lin N. H. Gatignol A. Glabe C. G. Fan H. (1993). In vitro and in vivo binding of human immunodeficiency virus type 1 Tat protein and Sp1 transcription factor. *J. Virol.* 67 6224–6233. 7690421 10.1128/jvi.67.10.6224-6233.1993 PMC238044

[B37] John M. Heckerman D. James I. Park L. P. Carlson J. M. Chopra A. (2010). Adaptive interactions between HLA and HIV-1: highly divergent selection imposed by HLA class I molecules with common supertype motifs. *J. Immunol.* 184 4368–4377. 10.4049/jimmunol.0903745 20231689 PMC3011274

[B38] Jones N. A. Wei X. Flower D. R. Wong M. Michor F. Saag M. S. (2004). Determinants of human immunodeficiency virus type 1 escape from the primary CD8+ cytotoxic T lymphocyte response. *J. Exp. Med.* 200 1243–1256. 10.1084/jem.20040511 15545352 PMC2211924

[B39] Kuang X. T. Li X. Anmole G. Mwimanzi P. Shahid A. Le A. Q. (2014). Impaired Nef function is associated with early control of HIV-1 viremia. *J. Virol.* 88 10200–10213. 10.1128/JVI.01334-14 24965469 PMC4136354

[B40] Kumar A. Abbas W. Herbein G. (2014). HIV-1 latency in monocytes/macrophages. *Viruses* 6 1837–1860. 10.3390/v6041837 24759213 PMC4014723

[B41] Kuppuswamy M. Subramanian T. Srinivasan A. Chinnadurai G. (1989). Multiple functional domains of Tat, the trans-activator of HIV-1, defined by mutational analysis. *Nucleic Acids Res.* 17 3551–3561. 10.1093/nar/17.9.3551 2542902 PMC317795

[B42] Lassen K. G. Ramyar K. X. Bailey J. R. Zhou Y. Siliciano R. F. (2006). Nuclear retention of multiply spliced HIV-1 RNA in resting CD4+ T cells. *PLoS Pathog.* 2:e68 10.1371/journal.ppat.0020068 PMC1487174 16839202

[B43] Li G. Piampongsant S. Faria N. R. Voet A. Pineda-Pena A. C. Khouri R. (2015). An integrated map of HIV genome-wide variation from a population perspective. *Retrovirology* 12:18 10.1186/s12977-015-0148-6 PMC4358901 25808207

[B44] Li J. Chen C. Ma X. Geng G. Liu B. Zhang Y. (2016). Long noncoding RNA NRON contributes to HIV-1 latency by specifically inducing tat protein degradation. *Nat. Commun.* 7:11730 10.1038/ncomms11730 PMC4909936 27291871

[B45] Lichterfeld M. Gandhi R. T. Simmons R. P. Flynn T. Sbrolla A. Yu X. G. (2012). Induction of strong HIV-1-specific CD4+ T-cell responses using an HIV-1 gp120/NefTat vaccine adjuvanted with AS02A in antiretroviral-treated HIV-1-infected individuals. *J. Acquir. Immune Defic. Syndr.* 59 1–9. 10.1097/QAI.0b013e3182373b77 21963936 PMC3241906

[B46] Lin X. Irwin D. Kanazawa S. Huang L. Romeo J. Yen T. S. (2003). Transcriptional profiles of latent human immunodeficiency virus in infected individuals: effects of Tat on the host and reservoir. *J. Virol.* 77 8227–8236. 10.1128/JVI.77.15.8227-8236.2003 12857891 PMC165222

[B47] Liu M. K. Hawkins N. Ritchie A. J. Ganusov V. V. Whale V. Brackenridge S. (2013). Vertical T cell immunodominance and epitope entropy determine HIV-1 escape. *J. Clin. Invest.* 123 380–393. 10.1172/JCI65330 23221345 PMC3533301

[B48] Liu Y. McNevin J. Zhao H. Tebit D. M. Troyer R. M. McSweyn M. (2007). Evolution of human immunodeficiency virus type 1 cytotoxic T-lymphocyte epitopes: fitness-balanced escape. *J. Virol.* 81 12179–12188. 10.1128/JVI.01277-07 17728222 PMC2169017

[B49] Liu Y. McNevin J. P. Holte S. McElrath M. J. Mullins J. I. (2011). Dynamics of viral evolution and CTL responses in HIV-1 infection. *PLoS ONE* 6:e15639 10.1371/journal.pone.0015639 PMC3024315 21283794

[B50] MacDougall T. H. Shattock R. J. Madsen C. Chain B. M. Katz D. R. (2002). Regulation of primary HIV-1 isolate replication in dendritic cells. *Clin. Exp. Immunol.* 127 66–71. 10.1046/j.1365-2249.2002.01715.x 11882034 PMC1906274

[B51] Marsden M. D. Zack J. A. (2015). Double trouble: HIV latency and CTL escape. *Cell Host Microbe* 17 141–142. 10.1016/j.chom.2015.01.008 25674977 PMC4375954

[B52] Marzio G. Tyagi M. Gutierrez M. I. Giacca M. (1998). HIV-1 tat transactivator recruits p300 and CREB-binding protein histone acetyltransferases to the viral promoter. *Proc. Natl. Acad. Sci. U.S.A.* 95 13519–13524. 10.1073/pnas.95.23.13519 9811832 PMC24851

[B53] Mason R. D. De Rose R. Kent S. J. (2009). Differential patterns of immune escape at Tat-specific cytotoxic T cell epitopes in pigtail macaques. *Virology* 388 315–323. 10.1016/j.virol.2009.03.020 19394064

[B54] Moreau E. Belliard G. Partidos C. D. Pradezinsky F. Le Buanec H. Muller S. (2004). Important B-cell epitopes for neutralization of human immunodeficiency virus type 1 Tat in serum samples of humans and different animal species immunized with Tat protein or peptides. *J. Gen. Virol.* 85(Pt 10), 2893–2901. 10.1099/vir.0.80365-0 15448351

[B55] Mousseau G. Kessing C. F. Fromentin R. Trautmann L. Chomont N. Valente S. T. (2015). The tat inhibitor Didehydro-Cortistatin A prevents HIV-1 reactivation from latency. *MBio* 6:e00465–15 10.1128/mBio.00465-15 PMC4495168 26152583

[B56] Mwimanzi P. Markle T. J. Martin E. Ogata Y. Kuang X. T. Tokunaga M. (2013). Attenuation of multiple Nef functions in HIV-1 elite controllers. *Retrovirology* 10:1 10.1186/1742-4690-10-1 PMC3564836 23289738

[B57] Neuveut C. Jeang K. T. (1996). Recombinant human immunodeficiency virus type 1 genomes with tat unconstrained by overlapping reading frames reveal residues in Tat important for replication in tissue culture. *J. Virol.* 70 5572–5581. 8764071 10.1128/jvi.70.8.5572-5581.1996 PMC190517

[B58] Pagans S. Pedal A. North B. J. Kaehlcke K. Marshall B. L. Dorr A. (2005). SIRT1 regulates HIV transcription via Tat deacetylation. *PLoS Biol.* 3:e41 10.1371/journal.pbio.0030041 PMC546329 15719057

[B59] Parada C. A. Roeder R. G. (1996). Enhanced processivity of RNA polymerase II triggered by Tat-induced phosphorylation of its carboxy-terminal domain. *Nature* 384 375–378. 10.1038/384375a0 8934526

[B60] Pearson R. Kim Y. K. Hokello J. Lassen K. Friedman J. Tyagi M. (2008). Epigenetic silencing of human immunodeficiency virus (HIV) transcription by formation of restrictive chromatin structures at the viral long terminal repeat drives the progressive entry of HIV into latency. *J. Virol.* 82 12291–12303. 10.1128/JVI.01383-08 18829756 PMC2593349

[B61] Peloponese J. M. Jr. Collette Y. Gregoire C. Bailly C. Campese D. Meurs E. F. (1999). Full peptide synthesis, purification, and characterization of six Tat variants. Differences observed between HIV-1 isolates from Africa and other continents. *J. Biol. Chem.* 274 11473–11478. 10206951 10.1074/jbc.274.17.11473

[B62] Peterlin B. M. Brogie J. E. Price D. H. (2012). 7SK snRNA: a noncoding RNA that plays a major role in regulating eukaryotic transcription. *Wiley Interdiscip. Rev. RNA* 3 92–103. 10.1002/wrna.106 21853533 PMC3223291

[B63] Price D. A. Goulder P. J. Klenerman P. Sewell A. K. Easterbrook P. J. Troop M. (1997). Positive selection of HIV-1 cytotoxic T lymphocyte escape variants during primary infection. *Proc. Natl. Acad. Sci. U.S.A.* 94 1890–1895. 10.1073/pnas.94.5.1890 9050875 PMC20013

[B64] Rana T. M. Jeang K. T. (1999). Biochemical and functional interactions between HIV-1 Tat protein and TAR RNA. *Arch. Biochem. Biophys.* 365 175–185. 10.1006/abbi.1999.1206 10328810

[B65] Ranasinghe S. Cutler S. Davis I. Lu R. Soghoian D. Z. Qi Y. (2013). Association of HLA-DRB1-restricted CD4(+) T cell responses with HIV immune control. *Nat. Med.* 19 930–933. 10.1038/nm.3229 23793098 PMC3974408

[B66] Razooky B. S. Pai A. Aull K. Rouzine I. M. Weinberger L. S. (2015). A hardwired HIV latency program. *Cell* 160 990–1001. 10.1016/j.cell.2015.02.009 25723172 PMC4395878

[B67] Rossenkhan R. Novitsky V. Sebunya T. K. Musonda R. Gashe B. A. Essex M. (2012). Viral diversity and diversification of major non-structural genes vif, vpr, vpu, tat exon 1 and rev exon 1 during primary HIV-1 subtype C infection. *PLoS ONE* 7:e35491 10.1371/journal.pone.0035491 PMC3348911 22590503

[B68] Roy C. N. Khandaker I. Furuse Y. Oshitani H. (2015a). Molecular characterization of full-length Tat in HIV-1 subtypes B and C. *Bioinformation* 11 151–160. 10.6026/97320630011151 25914449 PMC4403036

[B69] Roy C. N. Khandaker I. Oshitani H. (2015b). Intersubtype genetic variation of HIV-1 Tat Exon 1. *AIDS Res. Hum. Retroviruses* 31 641–648. 10.1089/AID.2014.0346 25748226

[B70] Roy S. Delling U. Chen C. H. Rosen C. A. Sonenberg N. (1990). A bulge structure in HIV-1 TAR RNA is required for Tat binding and Tat-mediated trans-activation. *Genes Dev.* 4 1365–1373. 10.1101/gad.4.8.1365 2227414

[B71] Ruben S. Perkins A. Purcell R. Joung K. Sia R. Burghoff R. (1989). Structural and functional characterization of human immunodeficiency virus tat protein. *J. Virol.* 63 1–8. 2535718 10.1128/jvi.63.1.1-8.1989 PMC247650

[B72] Ruelas D. S. Greene W. C. (2013). An integrated overview of HIV-1 latency. *Cell* 155 519–529. 10.1016/j.cell.2013.09.044 24243012 PMC4361081

[B73] Rusnati M. Tulipano G. Spillmann D. Tanghetti E. Oreste P. Zoppetti G. (1999). Multiple interactions of HIV-I Tat protein with size-defined heparin oligosaccharides. *J. Biol. Chem.* 274 28198–28205. 10.1074/jbc.274.40.28198 10497173

[B74] Schellens I. M. Kesmir C. Miedema F. van Baarle D. Borghans J. A. (2008). An unanticipated lack of consensus cytotoxic T lymphocyte epitopes in HIV-1 databases: the contribution of prediction programs. *AIDS* 22 33–37. 10.1097/QAD.0b013e3282f15622 18090389

[B75] Smith S. M. Pentlicky S. Klase Z. Singh M. Neuveut C. Lu C. Y. (2003). An in vivo replication-important function in the second coding exon of Tat is constrained against mutation despite cytotoxic T lymphocyte selection. *J. Biol. Chem.* 278 44816–44825. 10.1074/jbc.M307546200 12947089

[B76] Tong-Starksen S. E. Baur A. Lu X. B. Peck E. Peterlin B. M. (1993). Second exon of Tat of HIV-2 is required for optimal trans-activation of HIV-1 and HIV-2 LTRs. *Virology* 195 826–830. 10.1006/viro.1993.1438 8337847

[B77] Troyer R. M. McNevin J. Liu Y. Zhang S. C. Krizan R. W. Abraha A. (2009). Variable fitness impact of HIV-1 escape mutations to cytotoxic T lymphocyte (CTL) response. *PLoS Pathog.* 5:e1000365 10.1371/journal.ppat.1000365 PMC2659432 19343217

[B78] Valentin A. Rosati M. Patenaude D. J. Hatzakis A. Kostrikis L. G. Lazanas M. (2002). Persistent HIV-1 infection of natural killer cells in patients receiving highly active antiretroviral therapy. *Proc. Natl. Acad. Sci. U.S.A.* 99 7015–7020. 10.1073/pnas.102672999 12011460 PMC124520

[B79] Verhoef K. Koper M. Berkhout B. (1997). Determination of the minimal amount of Tat activity required for human immunodeficiency virus type 1 replication. *Virology* 237 228–236. 10.1006/viro.1997.8786 9356335

[B80] Watkins J. D. Lancelot S. Campbell G. R. Esquieu D. de Mareuil J. Opi S. (2006). Reservoir cells no longer detectable after a heterologous SHIV challenge with the synthetic HIV-1 Tat Oyi vaccine. *Retrovirology* 3:8 10.1186/1742-4690-3-8 PMC1434768 16441880

[B81] Weeks K. M. Crothers D. M. (1991). RNA recognition by Tat-derived peptides: interaction in the major groove? *Cell* 66 577–588. 10.1016/0092-8674(81)90020-9 1907891

[B82] Wei P. Garber M. E. Fang S. M. Fischer W. H. Jones K. A. (1998). A novel CDK9-associated C-type cyclin interacts directly with HIV-1 Tat and mediates its high-affinity, loop-specific binding to TAR RNA. *Cell* 92 451–462. 10.1016/S0092-8674(00)80939-3 9491887

[B83] Westrop S. J. Qazi N. A. Pido-Lopez J. Nelson M. R. Gazzard B. Gotch F. M. (2009). Transient nature of long-term nonprogression and broad virus-specific proliferative T-cell responses with sustained thymic output in HIV-1 controllers. *PLoS ONE* 4:e5474 10.1371/journal.pone.0005474 PMC2677159 19434236

[B84] Yedavalli V. S. Benkirane M. Jeang K. T. (2003). Tat and trans-activation-responsive (TAR) RNA-independent induction of HIV-1 long terminal repeat by human and murine cyclin T1 requires Sp1. *J. Biol. Chem.* 278 6404–6410. 10.1074/jbc.M209162200 12458222

[B85] Yukl S. Pillai S. Li P. Chang K. Pasutti W. Ahlgren C. (2009). Latently-infected CD4+ T cells are enriched for HIV-1 Tat variants with impaired transactivation activity. *Virology* 387 98–108. 10.1016/j.virol.2009.01.013 19268337 PMC4474533

[B86] Yusim K. Kesmir C. Gaschen B. Addo M. M. Altfeld M. Brunak S. (2002). Clustering patterns of cytotoxic T-lymphocyte epitopes in human immunodeficiency virus type 1 (HIV-1) proteins reveal imprints of immune evasion on HIV-1 global variation. *J. Virol.* 76 8757–8768. 10.1128/JVI.76.17.8757-8768.2002 12163596 PMC136996

[B87] Zhai S. Zhuang Y. Song Y. Li S. Huang D. Kang W. (2008). HIV-1-specific cytotoxic T lymphocyte (CTL) responses against immunodominant optimal epitopes slow the progression of AIDS in China. *Curr. HIV Res.* 6 335–350. 10.2174/157016208785132473 18691032

